# Research Protocol for an Observational Health Data Analysis on the Adverse Events of Systemic Treatment in Patients with Metastatic Hormone-sensitive Prostate Cancer: Big Data Analytics Using the PIONEER Platform

**DOI:** 10.1016/j.euros.2024.02.019

**Published:** 2024-03-25

**Authors:** Pawel Rajwa, Angelika Borkowetz, Thomas Abbott, Andrea Alberti, Anders Bjartell, James T. Brash, Riccardo Campi, Andrew Chilelli, Mitchell Conover, Niculae Constantinovici, Eleanor Davies, Bertrand De Meulder, Sherrine Eid, Mauro Gacci, Asieh Golozar, Haroon Hafeez, Samiul Haque, Ayman Hijazy, Tim Hulsen, Andreas Josefsson, Sara Khalid, Raivo Kolde, Daniel Kotik, Samu Kurki, Mark Lambrecht, Chi-Ho Leung, Julia Moreno, Rossella Nicoletti, Daan Nieboer, Marek Oja, Soundarya Palanisamy, Peter Prinsen, Christian Reich, Giulio Raffaele Resta, Maria J. Ribal, Juan Gómez Rivas, Emma Smith, Robert Snijder, Carl Steinbeisser, Frederik Vandenberghe, Philip Cornford, Susan Evans-Axelsson, James N'Dow, Peter-Paul M. Willemse

**Affiliations:** a Department of Urology, Medical University of Silesia, Zabrze, Poland; b Department of Urology, Comprehensive Cancer Center, Medical University of Vienna, Vienna, Austria; c Department of Urology, University Hospital Carl Gustav Carus, TU Dresden, Dresden, Germany; d European Association of Urology, Nijmegen, The Netherlands; e Unit of Urological Robotic Surgery and Renal Transplantation, University of Florence, Careggi Hospital, Florence, Italy; f Department of Translational Medicine, Lund University, Lund, Sweden; g IQVIA, Real World Solutions, Brighton, UK; h Astellas Pharma Europe Ltd, Surrey, UK; i Janssen Research & Development, Titusville, NJ, USA; j Bayer AG, Berlin, Germany; k Association EISBM, Vourles, France; l SAS Institute, Cary, NC, USA; m Odysseus Data Services, New York, NY, USA; n OHDSI Center, Northeastern University, Boston, MA, USA; o Shaukat Khanum Memorial Cancer Hospital & Research Centre, Peshawar, Pakistan; p Department of Hospital Services & Informatics, Philips Research, Eindhoven, The Netherlands; q Department of Urology, Institute of Clinical Sciences, Sahlgrenska Academy, University of Gothenburg, Gothenburg, Sweden; r Wallenberg Center for Molecular Medicine, Umeå University, Umeå, Sweden; s University of Oxford, Oxford, UK; t Institute of Computer Science, University of Tartu, Tartu, Estonia; u Center for Advanced Systems Understanding, Görlitz, Germany; v Helmholtz-Zentrum Dresden-Rossendorf, Dresden, Germany; w Bayer OY, Turku, Finland; x S.H. Ho Urology Centre, Department of Surgery, The Chinese University of Hong Kong, Hong Kong, China; y Erasmus MC University Medical Center, Rotterdam, The Netherlands; z Netherlands Comprehensive Cancer Organisation (IKNL), Utrecht, The Netherlands; aa Uro-Oncology Unit, Hospital Clinic, University of Barcelona, Barcelona, Spain; bb Department of Urology, Hospital Clinico San Carlos, Madrid, Spain; cc Guidelines Office, European Association of Urology, Arnhem, The Netherlands; dd Collaborate Project Management, Munich, Germany; ee Liverpool University Hospitals NHS Trust, Liverpool, UK; ff Academic Urology Unit, University of Aberdeen, Aberdeen, UK; gg Department of Urology, Cancer Center, University Medical Center Utrecht, Utrecht, The Netherlands

**Keywords:** Prostate cancer, Metastatic, Hormone sensitive, Docetaxel, Androgen receptor signaling inhibitor, Big data, PIONEER

## Abstract

Combination therapies in metastatic hormone-sensitive prostate cancer (mHSPC), which include the addition of an androgen receptor signaling inhibitor and/or docetaxel to androgen deprivation therapy, have been a game changer in the management of this disease stage. However, these therapies come with their fair share of toxicities and side effects. The goal of this observational study is to report drug-related adverse events (AEs), which are correlated with systemic combination therapies for mHSPC. Determining the optimal treatment option requires large cohorts to estimate the tolerability and AEs of these combination therapies in “real-life” patients with mHSPC, as provided in this study. We use a network of databases that includes population-based registries, electronic health records, and insurance claims, containing the overall target population and subgroups of patients defined by unique certain characteristics, demographics, and comorbidities, to compute the incidence of common AEs associated with systemic therapies in the setting of mHSPC. These data sources are standardised using the Observational Medical Outcomes Partnership Common Data Model. We perform the descriptive statistics as well as calculate the AE incidence rate separately for each treatment group, stratified by age groups and index year. The time until the first event is estimated using the Kaplan-Meier method within each age group. In the case of episodic events, the anticipated mean cumulative counts of events are calculated. Our study will allow clinicians to tailor optimal therapies for mHSPC patients, and they will serve as a basis for comparative method studies.

## Take Home Message

Despite their efficacy, novel combination systemic therapies for metastatic hormone-sensitive prostate cancer (mHSPC) are associated with side effects. Our study reports drug-related adverse events in a large cohort of “real-life” patients treated with combination therapies for mHSPC.

## 1 Introduction

The treatment of metastatic hormone-sensitive prostate cancer (mHSPC) has evolved over time with the introduction of combination systemic therapies, which are more effective than androgen deprivation therapy (ADT) alone [1]. These combination therapies include ADT in addition to either chemotherapy (docetaxel) or an androgen receptor signaling inhibitor (ARSI; enzalutamide, apalutamide, abiraterone acetate, or darolutamide) or both (triplet therapy) [1], [2], [3]. The PEACE-1 and ARASENS trials showed a benefit of ARSI addition to docetaxel plus ADT [4], [5]. However, none of the existing trials can answer which of the doublet combination therapies is associated with the highest efficacy. Furthermore, in recent network meta-analyses, none of the doublets was significantly superior to the other in terms of efficacy [3], and triplet therapy outperformed doublets only in selected subgroups [1], [2], albeit also associated with a higher risk of severe adverse events (AEs).

Each of the novel therapies comes with their own set of toxicities and AEs [1]. For example, combination therapies are associated with a higher risk of cardiovascular [1], cognitive [2], [6], and nervous system toxicity [2], as well as fatigue [1] compared with ADT monotherapy. Therefore, the key considerations in the management of mHSPC become safety and toxicity profiles of available combinations. In other words, the pivotal question is whether the patient can tolerate selected combination as opposed to alternative treatment with better safety and quality of life. Furthermore, in patients with multiple comorbidities, introduction of more drugs at baseline can develop drug-drug and drug-condition interactions, leading to increased AEs and hospitalisations. All this increases the need for selecting the right therapy for the right patient at the right time.

Overall, previous reports show that registry-based real-world data on AEs differ from those in clinical trial setting [7], [8]. Most of the real-world evidence on ARSI and docetaxel combination is derived from metastatic castration-resistant prostate cancer (mCRPC) patients [9], [10], [11]. For example, Conover et al [12] showed in the US administrative claims data that in the mCRPC setting, abiraterone acetate is associated with a higher risk of heart failure, acute myocardial infarction, and ischemic stroke than enzalutamide. In addition, Bjartell et al [9] found that in patients treated with hormonal and chemotherapy sequencing for mCRPC, drug toxicity was the second most common cause of treatment discontinuation. However, there are little to no data on the safety and incidence of AEs in patients treated with combination therapies for mHSPC outside clinical trials. Therefore, we aimed to characterise and report the incidence of AEs in a large cohort of patients undergoing treatment for mHSPC using approved combination therapies.

## 2 Design

This is an observational study to report the rate of prespecified AEs among new mHSPC users of ADT monotherapy, ARSI, docetaxel, or docetaxel plus ARSI using the PIONEER platform [13], [14], [15], [16], [17], [18]. Definitions of key terms used in study are is included in Table 1. Proposed project-related studies are included in Supplementary Table 1.

**Table 1:** Definitions of key terms used in study protocol

Terms	Definition of terms
Index (cohort entry date)	Index per cohort will be defined as follows: ADT—first record of ADT after metastasis ARSI—first record of ARSI Docetaxel—first record of docetaxel ARSI plus docetaxel—first record of ARSI or docetaxel
Baseline period	Time prior to index for the following variables: 1. Metastases—90 d prior to index 2. PCa assessment—no limitation 3. Other baseline variables—365 d prior to index
Study period	No study period defined
Chronic AEs	AEs that a patient is assumed to have from their first record of the condition until the end of the observation period. Thus, in the analysis, only the first record of a chronic AE is considered
Chronic AEs of interest	Alopecia Chronic heart failure Cognitive disturbance Diabetes Hypertension Neuropathy Abnormal hepatic function Kidney failure
Episodic AE	AE for which every event is eligible for an analysis, providing the events are separated by a prespecified number of days
Episodic AE of interest	Acute cardiac event Bone marrow suppression Cerebral event Electrolyte imbalance Falls Fatigue GI toxicity Hospitalisation Sepsis Kidney failure Rash Seizures Skeletally related events Thromboembolism Abnormal hepatic function Kidney failure
Triple therapy	Concomitant administration of ARSI and docetaxel
Treatment duration	Continuous treatment was defined as follows: ADT—until the end of the observation period ARSI—until a gap of >60 d between records Docetaxel—until the end of the observation period
ARSI	abiraterone acetate, apalutamide, enzalutamide, and darolutamide

ADT = androgen deprivation therapy; AE = adverse event; ARSI = androgen receptor signalling inhibitor; PCa = prostate cancer.

### 2.1 Objectives

Overall, this study aims to characterise and report the incidence of drug-associated AEs in the following four cohorts over defined follow-up: ADT monotherapy, ARSI, docetaxel, and ARSI plus docetaxel in real-world patients treated for mHSPC (Table 2).

**Table 2:** Summary of objectives

Objectives	Endpoints
*Primary*	
Report AEs per cohort	Number of AEs of interest on treatment (Table 2) per cohort
*Secondary*	
Report time to AEs of interest per cohort	Episodic AEs: time from index date to first documentation of episodic AE of interest per cohort Chronic AEs: time from index to the documentation of chronic AE of interest per cohort
Follow-up time	Time from index date to end of observation (ADT-only and docetaxel cohorts), end of continuous ARSI use (ARSI cohorts), censoring event (treatment switch, diagnosis of other cancer), or loss to follow-up
Patients’ baseline characteristics of interest	Baseline characteristics defined by the following variables (where available): 1. Age in years 2. Index year 3. CCI algorithmic 4. Obesity a 5. Performance status 6. Individual treatment received within ARSI cohort 7. Individual treatment received within ARSI plus docetaxel cohort 8. Gleason score 9. Comorbidities of interest prior to index 10. Number of hospitalisations in the year prior to index 11. Time from index to intensification for doublet and triplet combination
Report hospitalisation associated with treatment	Number of hospitalisations of interest on treatment

AE = adverse events; ADT = androgen deprivation therapy; ARSI = androgen signalling inhibitor; CCI = Charlson Comorbidity Index.

a Obesity: diagnostic codes associated with obesity.

The primary objective is to report the incidences of AEs of interest among patients with mHSPC receiving ADT monotherapy, ADT plus ARSI, ADT plus docetaxel, or ADT in combination with ARSI and docetaxel.

The secondary objectives are as follows:

1. To analyse time to AEs associated with ARSI, docetaxel, and docetaxel plus ARSI for mHSPC
2. To report baseline characteristics of patients treated with ARSI, docetaxel, and docetaxel plus ARSI for mHSPC
3. To report hospitalisation rates for the ARSI, docetaxel, and docetaxel plus ARSI cohorts for mHSPC

## 3 Methods

### 3.1 Data sources

Electronic health records (EHRs), registries, and administrative claims databases all converted to the Observational Medical Outcomes Partnership (OMOP) Common Data Model will be utilised (Supplementary Table 2). This includes a standard representation of healthcare experiences (such as information related to drug utilisation and condition occurrence) as well as common vocabularies for coding clinical concepts, and enables consistent application of analyses across multiple disparate data (OHDSI, 2020; Voss, 2015). All analyses will be performed independently within each database to produce database-specific results for each analysis.

### 3.2 Target cohorts

The target cohorts for this study are mHSPC patients who were treated with ADT monotherapy, ARSI, docetaxel, or docetaxel plus ARSI. Cohorts will be indexed on the first eligible treatment recorded within a (-30, 90) d window relative to the earliest metastasis diagnosis. For the ADT monotherapy cohort, no details of docetaxel or ARSI may be recorded any time before and up to 183 d after index ADT, unless it follows a censoring event. A cut-off of 6 mo was selected to minimise the risk of treatment for localised disease or initial combination therapy, however still to capture treatment for progression on ADT. For the ARSI cohorts, no details of docetaxel may be recorded any time before and up to 183 d after index ARSI, unless it follows a censoring event. For the docetaxel cohort, no details of ARSI may be recorded any time before and up to 183 d after index docetaxel, unless it follows a censoring event. In addition, in the docetaxel cohort, patients on ADT who have a record of chemotherapy, but without a named drug reported, and no record of ARSI, 30 d before and up to 183 d after the index date will be included under the presumption that this chemotherapy would likely be docetaxel; in the ADT-only and ARSI plus ADT cohorts, this is an exclusion criterion. A sensitivity analysis will be performed to compare the cohort treated with docetaxel and the cohort with only the notification of chemotherapy treatment. For the docetaxel plus ARSI cohort, either docetaxel or ARSI may serve as the index event, with the alternative treatment having to occur within 183 d of the index treatment. The following operational definitions will be applied to identify mHSPC patients. Patients must have a record indicating metastases plus a record of prostate cancer (PCa) that occurs any time before or up to 30 d after the metastasis record. To ensure that metastases are due to PCa, patients must not have a record for any other primary cancer at any time before metastases, with the exception of nonmelanoma skin cancer (-∞ to +30 d). To identify mHSPC patients, patients must not have a record of orchiectomy >30 d before metastasis and they must not have a record of chemical ADT within a (-365 to -31) d window relative to metastasis.

**Fig. 1:**
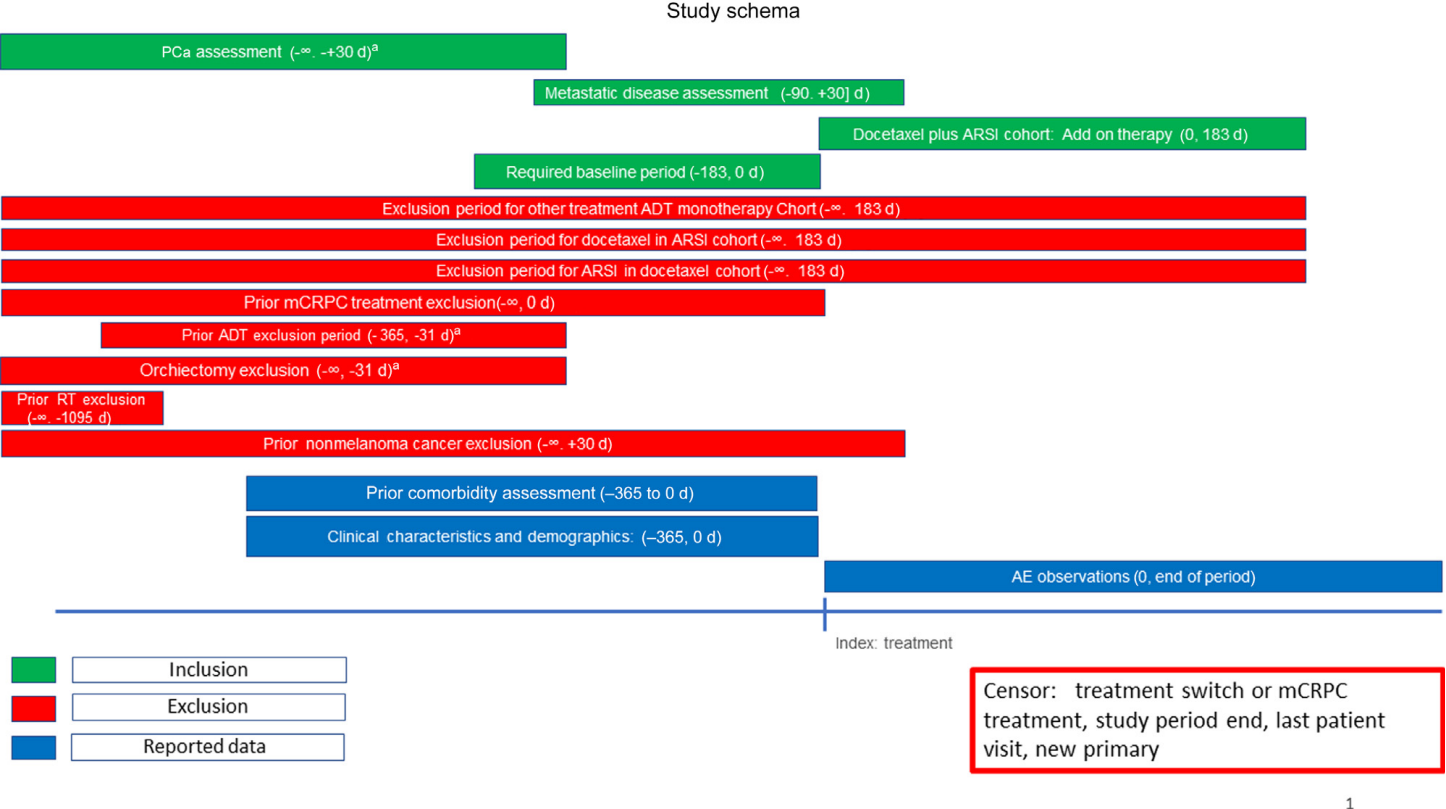
Study schema of PIONEER study-a-thon III. ADT = androgen deprivation therapy; AE = adverse event; ARSI = androgen receptor signalling inhibitor; mCRPC = metastatic castration-resistant prostate cancer; PCa = prostate cancer; RT = radiotherapy. ^a^ ADT and orchiectomy exclusion window and PCa assessment anchored to metastasis.

Index events, and inclusion and exclusion criteria of the target cohort (mHSPC patients treated with ADT monotherapy, ARSI, docetaxel, or ARSI plus docetaxel; Fig. 1) are detailed below.

#### 3.2.1 Index event

The earliest record of exposure for mHSPC to:

1. ADT if used as monotherapy
2. ARSI
3. Docetaxel without ARSI
4. Docetaxel with ARSI

#### 3.2.2 Inclusion criteria

1. Male adults (age ≥18 yr at index)
2. At least one diagnosis of metastatic disease; earliest diagnosis must be recorded within (-90 to +30 d) of index
3. At least one diagnosis of PCa, recorded within (-∞ to +30 d) of earliest diagnosis of metastases
4. No other primary cancer, except for nonmelanoma skin cancer, recorded within (-∞ to +30 d) of index
5. No chemical ADT recorded (-365 to -31 d) before the earliest diagnosis of metastases
6. No orchiectomy recorded (-∞ to -31 d) before the earliest diagnosis of metastases
7. No treatment exclusively indicated for mCRPC recorded any time before index (cabazitaxel, paclitaxel, lutetium-177, poly (ADP-ribose) polymerase inhibitors, immunotherapy, and radium-223)
8. No alternative index treatment recorded any time before index (with the exception of chemical ADT recorded >365 d before the earliest diagnosis of metastases)
9. No radiotherapy recorded 1095 d prior to index
10. For the ARSI, docetaxel, and docetaxel plus ARSI cohorts, an assumption is made that ADT is concomitantly required in each agent’s regulatory approval for mHSPC

#### 3.2.3 Follow up

Patient follow-up will be determined as follows:

1. Until the end of observation period for the ADT monotherapy and docetaxel index groups
2. Until a gap in ARSI records of >60 d for the ARSI and ARSI + docetaxel index groups

Patient follow-up will end in the event of one of the following censoring events:

1. Diagnosis of a primary cancer other than prostate or nonmelanoma skin cancer
2. Exposure to radiotherapy or a treatment exclusively indicated for mCRPC (cabazitaxel, paclitaxel, lutetium-177, poly [ADP-ribose] polymerase inhibitors, immunotherapy, and radium-223)
3. Exposure to an alternative index treatment group, including switching ARSIs; a record of ADT would not constitute a censoring event

### 3.3 Outcomes

The prespecified AEs of interest were determined during authors’ consensus meeting and based on AEs reported in main trials assessing the efficacy and safety of drugs in patients with mHSPC (Supplementary Table 3).

The following are the AEs of interest:

1. Abnormal hepatic function
2. Acute cardiac event
3. Alopecia
4. Bone marrow suppression
5. Cerebral event
6. Chronic heart failure including peripheral oedema
7. Cognitive disturbance
8. Diabetes
9. Electrolyte imbalance
10. Falls
11. Fatigue
12. Gastrointestinal toxicity
13. Hospitalisation
14. Hypertension
15. Sepsis
16. Kidney failure
17. Neuropathy
18. Rash
19. Seizures
20. Skeletally related events
21. Thromboembolism

### 3.4 Statistical analyses

#### 3.4.1 Sample size and power

The study will perform a descriptive analysis of the ADT monotherapy, ARSI, docetaxel, and docetaxel plus ARSI cohorts, and as such no minimum sample is required. For each analysis to be reported, the minimal number of patients/events required is 5.

#### 3.4.2 Stratifications

Each target cohort’s ADT monotherapy, ARSI, docetaxel, and docetaxel plus ARSI will be analysed in full and stratified on factors based on the following baseline characteristics assessed for the 1-yr preindex period; all strata are pending meeting minimum reportable cell counts (as specified by data owners).

The baseline characteristics are as follows:

1. Age at index (raw data)
2. Comorbidities reported separately and classified according to standardised systems (eg, Charlson Comorbidity Index [CCI]). Patients will be stratified into three groups:
  (a) CCI = 0
  (b) CCI = 1
  (c) CCI ≥2
3. Comorbidities recorded:
  (a) At any time point before the index date
  (b) 365 d before the index date
  (c) 30 d before the index date
  (d) At the index date

#### 3.4.3 Characterisation analysis

All analyses will be performed using the code developed and adapted from the OHDSI Methods library. The code for this analysis can be found at https://github.com/ahijazy/PioneerMetastaticAE. A single package executes cohort diagnostics to assess the fitness of use of the phenotypes in the database and the characterisation step. Baseline covariates will be extracted using an optimised SQL extraction script to quantify demographics, condition group eras, and drug group eras. Additional cohort-specific covariates will be constructed using OMOP Standard Vocabulary concepts [19].

At the time of execution, the package will create a data frame in which individuals’ age and sex will be extracted. Individuals’ medical conditions, procedures, measurements, and medications will be summarised over several time periods: all time (-∞ to +∞), all time prior (-∞ to 0), a year prior (-365 to 0), a year to a month prior (-365 to -31), 6 mo prior (-180 to 0), and a month prior (-31 to 0) the index date; at the index date; and over the follow-up period of 1 mo (0 to 31) and from 1 mo to a year (31 to 365). The numbers and proportions of persons with feature variables during time-at-risk (TAR) windows will be reported by the target cohort and specific stratifications. Standardised mean differences will be calculated when comparing the characteristics of study cohorts, with plots comparing the mean values of characteristics. Baseline disease characteristics at diagnostics will be reported using the median and proportions for non-normally distributed continuous variables and categorical variables, respectively. The median follow-up will be computed for the overall study cohort. The absolute number of patients who experienced each AE will be reported.

#### 3.4.4 Estimation analysis

The objective of this analysis is to estimate the crude incidence rates (per 1000 person-years) and incidence proportions (per 1000 persons) of AEs across the ADT monotherapy, ARSI, docetaxel, and docetaxel plus ARSI cohorts. In reporting estimated incidence rates per 1000 person-years rather than real numbers, we allow comparability across different populations and time periods. This approach adjusts for variations in population sizes and exposure times, ensuring a more accurate reflection of AE occurrences. Nevertheless, we report the number of as well as the rates in the Shiny app. Crude incidence rates will be estimated and stratified by the database. For the time-to-event analysis, Kaplan-Meier estimates of event-free survival for each database are evaluated. Furthermore, we will estimate regarding the TAR specifications for chronic and episodic events in the incidence rate analysis:

The chronic events are listed as follows:

1. Patients experiencing the AEs before the start of the TAR are excluded.
2. A record of a chronic event during the observation period ends the TAR, the event is counted in the incidence rate, and follow up time is the timespan between the cohort start date and the time of the occurrence of the chronic event.

The episodic events are the following:

1. Having the events prior to the TAR does not exclude the patients.

2. The follow-up time is the time span between the treatment cohort start date and the cohort end date. This is added up if there are multiple observation periods. All AE event occurrences during this TAR are counted in the incidence rate calculation.

It is important to note that the incidence analysis conducted is descriptive and univariate in nature, focusing on presenting and summarising the incidence rates of the AEs. It aimed to provide an overview and understanding of the occurrence of AEs within a target population. Therefore, comparing incidence rates directly is not appropriate in this context. Incidence rates can be influenced by various factors, such as population demographics or the presence of confounding variables. Hence, caution should be exercised when comparing incidence rates between different groups or time periods, as it may lead to misleading interpretations. A further analysis, such as a comparative effectiveness study with propensity score matching, is needed before comparisons can be made. Once sufficient data are available, we intend to conduct this study to explore and assess the comparative effectiveness of different treatments based on AEs, thus studying the safety profiles of various interventions, enabling us to make informed decisions and enhance patient outcomes. Another critical point is that the incidence rates being calculated are not limited to treatment-caused events but encompass all events experienced by the patients. On the one hand, this offers a more comprehensive view of their health outcomes. On the other hand, one cannot infer a direct cause-and-effect relationship between treatments and specific outcomes (AEs). In other words, it is not possible to determine which events are directly attributable to the treatment and which are not.

The third objective is to estimate the mean cumulative failure (MCF) of episodic events. MCF is a statistical measure used in survival analysis, particularly when assessing the occurrence of events or failures over time. It represents the cumulative number of events that will have occurred by a specific time point. MCF allows one to understand how the risk of the event changes over time and provides valuable insights into the occurrence of AEs under study. MCF will be estimated using the Nelson-Aalen estimator [18]. The Nelson-Aalen estimator, a nonparametric estimator in the point estimate of sample MCF at each time point, does not assume any underlying model.

#### 3.4.5 Sensitivity analysis

Castration sensitivity is central to the definition of mHSPC and relies on well-captured prior ADT use in data sets. However, we have a reason to believe that ADT may be under-reported in some data sources, a bias that may be significantly greater in the periods prior to obtaining specific indications for the mHSPC populations. To understand the risk of misclassification of mCRPC patients as mHSPC patients, an analysis will be undertaken to assess in the datasets whether ADT is associated with agents requiring it according to their label. Another sensitivity analysis will describe baseline characteristics and outcomes of cohorts when restrictions are placed to define time periods during which agents have received regulatory approval for mHSPC (Supplementary Table 4). The baseline analysis requires intensification with a period from index to 183 d with the intent to limit misclassification of mCRPC patients as mHSPC patients. While this approach supports target cohort specificity, it may limit generalisability of the findings to routine clinical practice. An analysis will be performed where the intensification period may be up to 90 d to reflect more general clinical practice. The baseline analysis requires index treatment to occur within a window of 30 d before and 90 d after the earliest record of metastases. An analysis will be performed where index treatment will be required to occur within 30 d of metastases.

#### 3.4.6 Logistics of executing a federated analysis

We will use both centrally hosted and federated datasets to perform this analysis. The analytics will be developed using readily available data, either centrally hosted or directly accessible by study-a-thon members. Once we reach a shareable version of the analytics package, we will circulate the link to the GitHub hosting the package, which will be downloaded by all data owners and run locally. We will then gather and collate the results in an online Shiny app available to all.

## 4 Strengths and limitations

### 4.1 Strengths

The study is anticipated to be the largest patient-level cohort of metastatic PCa patients, thus allowing characterisation of relatively uncommon outcomes otherwise not identifiable in smaller datasets. Data will be obtained from multiple centres and providers. The data sets also represent patient-level data from multiple countries, which aids in the generalisability of findings. This enables comprehensive characterisation of the study population, key baseline characteristics, and outcomes. Several sensitivity analyses have been conducted, which will aid in the interpretation and lend robustness to results. Lastly, the use of routinely collected data from multiple sources maximises the external validity and generalisability of the findings.

### 4.2 Limitations

This study is carried out using data recorded in a collection of EHRs, claims, and tumour registries. As with any healthcare database used for a secondary data analysis, the patient records might be incomplete in many respects and may have had erroneous entries, leading to misclassification of study variables. Data regarding diagnosis of metastatic PCa, treatments, pathology, imaging, and laboratory results or baseline covariates prior to enrolment within the database may not be available. Clinical progression based on radiological imaging is limited by the data collection. PCa-specific characteristics such as stage, grade at diagnosis, or the extent of the disease are not readily available in most EHRs and claims databases. A selection bias cannot be ruled out as patient factors such as age, comorbidities, and clinical characteristics may influence treatment choice and subsequent outcomes. Treatment provided in hospitals or any other setting outside each participating institution is not included. Medical conditions may be underestimated as these will be based on the presence of condition codes, with the absence of such a record taken to indicate the absence of a disease. Meanwhile, medication records indicate that an individual was prescribed or dispensed a particular drug, but this does not necessarily mean that an individual took the drug as originally prescribed or dispensed. In the real world, there does not exist consistent documentation of AEs that we see in trials using a system such as Common Terminology Criteria for Adverse Events. Additionally, cohorts have not been matched or weighted to ensure comparable groups at baseline.

## 5 Protection of human individuals

The study uses only deidentified data. Confidentiality of patient records will be maintained at all times. Data custodians will remain in full control of executing the analysis and packaging results. There will be no transmission of patient-level data at any time during these analyses. Only aggregate statistics will be captured. Study packages will contain minimum cell count parameters to obscure any cells that fall below the allowable reportable limits. All study reports will contain aggregate data only and will not identify individual patients or physicians.

## 6 Management and reporting of AEs and adverse reactions

According to the new guidelines for good pharmacovigilance practice (EMA/873138/2011) and ISPE, there is no requirement for expedited reporting of adverse drug reactions from studies with secondary use of data (such as electronic healthcare databases).

## 7 Plans for disseminating and communicating study results

The results of the study will be presented at international urological and oncological meetings in the form of abstracts. The final results will be published as full-text papers in an international peer-reviewed urological journal. The results of this study will be published following guidelines, including those for authorship, established by the International Committee of Medical Journal Editors. When reporting results of this study, the appropriate Strengthening the Reporting of Observational Studies in Epidemiology checklist will be followed.

## 8 Sponsor

The study is supported by the Innovative Medicines Initiative 2 (IMI2) Joint Undertaking project PIONEER (grant agreement no. 777492) [13], [14], [15], [16], [17], [18]. IMI2 receives support from the European Union’s Horizon 2020 research programme and the European Federation of Pharmaceutical Industries and Associations (EFPIA). Many of the contributors are part of OHDSI, a multistakeholder interdisciplinary collaborative to bring out the value of health data through large-scale analytics, and may have other funding sources, which will be listed in the study manuscripts.

  ***Author contributions*:** Pawel Rajwa had full access to all the data in the study and takes responsibility for the integrity of the data and the accuracy of the data analysis.

  *Study concept and design*: Rajwa, Borkowetz, Abbott, Alberti, Bjartell, Brash, Chilelli, Constantinovici, Davies, De Meulder, Gacci, Golozar, Hijazy, Rivas, Cornford, Evans-Axelsson, Willemse.

*Acquisition of data*: Campi, Brash, De Meulder, Conover, Hafeez, Hijazy, Khalid, Leung, Nicoletti, Nieboer, Oja, Prinsen, Resta, Snijder.

*Analysis and interpretation of data*: Rajwa, Brash, Davies, Golozar, Hijazy, Oja, Prinsen, Willemse.

*Drafting of the manuscript*: Rajwa, Abbott, Brash, Chilelli, Hijazy, Evans-Axelsson, Willemse.

*Critical revision of the manuscript for important intellectual content*: Josefsson, Hulsen, Reich, Ribal.

*Statistical analysis*: Brash, Davies, De Meulder, Hijazy, Kolde, Kotik, Eid, Haque, Lambrecht, Moreno, Palanisamy, Vandenberghe.

*Obtaining funding*: Smith, N’Dow.

*Administrative, technical, or material support*: Brash, De Meulder, Hijazy, Kotik, Kurki, Smith, Steinbeisser.

*Supervision*: N’Dow, Cornford, Willemse.

*Other*: None.

  ***Financial disclosures:*** Pawel Rajwa certifies that all conflicts of interest, including specific financial interests and relationships and affiliations relevant to the subject matter or materials discussed in the manuscript (eg, employment/affiliation, grants or funding, consultancies, honoraria, stock ownership or options, expert testimony, royalties, or patents filed, received, or pending), are the following: None.

  ***Funding/Support and role of the sponsor*:** PIONEER is funded through the IMI2 Joint Undertaking and is listed under grant agreement no. 777492. This joint undertaking receives support from the European Union’s Horizon 2020 research and innovation programme, and European Federation of Pharmaceutical Industries and Associations (EFPIA). The EHDEN has received funding from the IMI2 Joint Undertaking under grant agreement no. 806968. The joint undertaking is supported by the European Union’s Horizon 2020 research and innovation programme and EFPIA, a large association that represents the biopharmaceutical industry in Europe. The views communicated within are those of PIONEER. None of the IMI, European Union, EFPIA, or any associated partners is responsible for any use that may be made of the information contained herein.
