# Supplementary material for: Research Protocol for an Observational Health Data Analysis on the Adverse Events of Systemic Treatment in Patients with Metastatic Hormone-sensitive Prostate Cancer: Big Data Analytics Using the PIONEER Platform
Source: Eur Urol Open Sci. 2024 Mar 25;63:81–8. doi: 10.1016/j.euros.2024.02.019 (PMC10987796; doi:10.1016/j.euros.2024.02.019)
Supplement: Supplementary data 1 [file mmc1.docx]

**Supplementary Table 1.** Prosposed project-related studies.

| **Principal Investigator/Author** | **Institution/Affiliation** | **Topics** |
| --- | --- | --- |
| Pawel Rajwa | Department of Urology, Medical University of Silesia, Zabrze, Poland; Department of Urology, Medical University of Vienna, Vienna Austria | Incidence of adverse events among patients treated with combination therapies for metastatic hormone-sensitive prostate cancer |
| Peter-Paul Willemse | Dept. Of Urology, Cancer Center, University Medical Center Utrecht, The Netherlands |  |
| Pawel Rajwa | Department of Urology, Medical University of Silesia, Zabrze, Poland; Department of Urology, Medical University of Vienna, Vienna Austria | Propensity-Score matched comparison of adverse events among patients treated with combination therapies for metastatic hormone-sensitive prostate cancer |
| Peter-Paul Willemse | Dept. Of Urology, Cancer Center, University Medical Center Utrecht, The Netherlands |  |
| Pawel Rajwa | Department of Urology, Medical University of Silesia, Zabrze, Poland; Department of Urology, Medical University of Vienna, Vienna Austria | Adverse Events in Metastatic Hormone-Sensitive Prostate Cancer Patients with Specific Comorbid Conditions Receiving Combination Treatments |
| Peter-Paul Willemse | Dept. Of Urology, Cancer Center, University Medical Center Utrecht, The Netherlands |  |
| Ayman Hijazy | European Institute for Systems Biology and Medicine | Methods for Defining Metastatic Hormone Sensitive Prostate Cancer in Patients Treated with ADT and Combination Therapies for mHSPC with OMOP Data: A PIONEER Project |
| Tom Abbott | European Association of Urology |  |
| Andrew Chilelli | HEOR Oncology, Astellas Pharma Europe Ltd., Surrey, UK |  |
