# Supplementary material for: Research Protocol for an Observational Health Data Analysis on the Adverse Events of Systemic Treatment in Patients with Metastatic Hormone-sensitive Prostate Cancer: Big Data Analytics Using the PIONEER Platform
Source: Eur Urol Open Sci. 2024 Mar 25;63:81–8. doi: 10.1016/j.euros.2024.02.019 (PMC10987796; doi:10.1016/j.euros.2024.02.019)
Supplement: Supplementary data 2 [file mmc2.docx]

**Supplementary Table 2.** Databases converted to the OMOP CDM with Prostate Cancer patients to be included in the PIONEER study-a-thon

| **Database Name/Center** | **Country** |
| --- | --- |
| Active Biotech | Sweden |
| CPRD | United Kingdom |
| DIAMOND | United Kingdom |
| ERSPC Rotterdam | Netherlands |
| Estonian biobank | Estonia |
| FinOMOP | Finland |
| FlatIron | United States of America |
| Florence University | Italy |
| Freiburg University | Germany |
| IQVIA AmbEMR | United States of America |
| IQVIA OncoEMR | United States of America |
| IQVIA Open Claims | United States of America |
| IQVIA Pharmetrics plus | United States of America |
| JANSSEN Prostate Cancer registry | International, Europe |
| MAITT | Estonia |
| MarketScan Merged | United States of America |
| Martini Klinik | Germany |
| MIRROR | Italy |
| Munich University | Germany |
| Netherlands Cancer Registry | Netherlands |
| OPTUM claims | United States of America |
| Teschnische Universität Dresden | Germany |
