# Supplementary material for: Research Protocol for an Observational Health Data Analysis on the Adverse Events of Systemic Treatment in Patients with Metastatic Hormone-sensitive Prostate Cancer: Big Data Analytics Using the PIONEER Platform
Source: Eur Urol Open Sci. 2024 Mar 25;63:81–8. doi: 10.1016/j.euros.2024.02.019 (PMC10987796; doi:10.1016/j.euros.2024.02.019)
Supplement: Supplementary data 3 [file mmc3.docx]

**Supplementary Table 3.** Toxicities of each combination therapies specified in the main manuscript bodies in the leading randomised controlled trials.

| Treatment | Study | Reported adverse events in the main manuscript body (of interest) |
| --- | --- | --- |
| ADT plus docetaxel | GETUG [1] | Alopecia, anaemia, constipation, decreased libido, diarrhoea, dyspnoea erectile dysfunction, fatigue, febrile neutropenia, gynaecomastia, hot flushes, increased ALT, increased AST, infections with neutropenia, mucositis, nail changes, nausea, neutropenia, peripheral oedema, sensory neuropathy, stomatitis, thrombocytopenia, vomiting |
|  | CHAARTED [2] | Allergic reaction, fatigue, diarrhoea, stomatitis, neuropathy, thromboembolism, sudden death, anaemia, thrombocytopenia, neutropenia, febrile neutropenia, infection with neutropenia |
|  | STAMPEDE [3] | Endocrine disorder, impotence, hot flushes, febrile neutropenia, neutropenia, general disorder, including lethargy, fever, asthenia, musculoskeletal adverse event, bone pain, generalised pain, gastrointestinal disorder, diarrhoea, abdominal pain, constipation, vomiting, renal adverse event, renal impairment, urinary-tract infection, respiratory disorder, dyspnoea, upper respiratory tract infection, cardiac disorder, hypertension, myocardial infarction, osteonecrosis of the jaw, nervous system disorders, peripheral neuropathy, nail changes |
| ADT plus enzalutamide | ARCHES [4] | Hot flushes, fatigue, arthralgia, back pain, increased weight, hypertension, diarrhoea, peripheral oedema, nausea, asthenia, constipation, musculoskeletal pain, dizziness, convulsion, hypertension, neutropenia, cognitive/memory impairment, ischemic heart disease, cardiovascular adverse events, posterior reversible encephalopathy syndrome, fatigue, fall, fractures, loss of consciousness, thrombocytopenia, musculoskeletal events, severe cutaneous adverse reactions, angioedema, rash, secondary primary malignancies |
|  | ENZAMET [5] | Febrile neutropenia, hypertension, neutropenia, fatigue, syncope, surgical or medical procedure, anaemia, fall, thromboembolic event, acute coronary syndrome, myocardial infarction, chest pain from cardiac cause, stroke, seizure, delirium |
| ADT plus abiraterone acetate | STAMPEDE [6] | Endocrine disorders, hypertension, myocardial infarction, cardiac dysrhythmia, musculoskeletal disorders, gastrointestinal disorders, hepatic disorders, increased AST, increased ALT, fatigue, oedema, respiratory disorders, dyspnoea, laboratory abnormalities, hypokalaemia |
|  | LATITUDE [7] | Back pain, hypertension, hot flush, arthralgia, hypokalaemia, fatigue,  increased AST, increased ALT, constipation, pain in extremity, peripheral oedema, bone pain, hyperglycaemia, anaemia, increased ldh, spinal cord compression, urinary retention, pneumonia, haematuria, cataract,  urinary tract infection, weight increased, hyperkalaemia, dyspnoea,  syncope, general physical health deterioration, hypertension, muscular weakness, musculoskeletal pain, osteonecrosis of jaw, asthenia, dysuria,  pathological fracture, nausea, vomiting, spinal pain, diabetes, angina pectoris, neutropenia, leucocytosis, deep vein thrombosis, pyrexia, decreased appetite, urinary tract obstruction, acute kidney injury, pulmonary embolism, platelet count decreased, neck pain, increased blood ALP, increased blood creatinine, abdominal pain, paraparesis, hepatic function disorder, dental caries, spinal compression fracture, thrombocytopenia, pain, tumour pain, gastritis, osteomyelitis acute |
| ADT plus apalutamide | TITAN [8] | Hot flushes, fatigue, hypertension, back pain, arthralgia, pain in arm and leg, pruritus, weight increased, anaemia, constipation, asthenia, bone pain, rash, increased blood ALP, urinary retention, rash, fall, fracture, hypothyroidism, seizures |
| Abiraterone triplet | PEACE-1 [9] | Hypertension, neutropenia, hepatotoxicity, febrile neutropenia, increased GTP, erectile dysfunction, increased ALP, fatigue, peripheral neuropathy |
| Darolutamide triplet | ARASENS [10] | Neutropenia, febrile neutropenia, hypertension, anaemia, pneumonia, hyperglycaemia, increased ALT, increased AST, increased weight, urinary tract infection |

1. Gravis G, Fizazi K, Joly F, Oudard S, Priou F, Esterni B, Latorzeff I, Delva R, Krakowski I, Laguerre B *et al*: **Androgen-deprivation therapy alone or with docetaxel in non-castrate metastatic prostate cancer (GETUG-AFU 15): A randomised, Open-label, Phase 3 trial**. *The Lancet Oncology* 2013, **14**(2):149-158.

2. Sweeney CJ, Chen YH, Carducci M, Liu G, Jarrard DF, Eisenberger M, Wong YN, Hahn N, Kohli M, Cooney MM *et al*: **Chemohormonal therapy in metastatic hormone-sensitive prostate cancer**. *New England Journal of Medicine* 2015, **373**(8):737-746.

3. James ND, Sydes MR, Clarke NW, Mason MD, Dearnaley DP, Spears MR, Ritchie AWS, Parker CC, Russell JM, Attard G *et al*: **Addition of docetaxel, zoledronic acid, or both to first-line long-term hormone therapy in prostate cancer (STAMPEDE): Survival results from an adaptive, multiarm, multistage, platform randomised controlled trial**. *The Lancet* 2016, **387**(10024):1163-1177.

4. Armstrong AJ, Szmulewitz RZ, Petrylak DP, Holzbeierlein J, Villers A, Azad A, Alcaraz A, Alekseev B, Iguchi T, Shore ND *et al*: **Arches: A randomized, phase III study of androgen deprivation therapy with enzalutamide or placebo in men with metastatic hormone-sensitive prostate cancer**. *Journal of Clinical Oncology* 2019, **37**(32):2974-2986.

5. Davis ID, Martin AJ, Stockler MR, Begbie S, Chi KN, Chowdhury S, Coskinas X, Frydenberg M, Hague WE, Horvath LG *et al*: **Enzalutamide with Standard First-Line Therapy in Metastatic Prostate Cancer**. *New England Journal of Medicine* 2019, **381**(2):121-131.

6. James ND, De Bono JS, Spears MR, Clarke NW, Mason MD, Dearnaley DP, Ritchie AWS, Amos CL, Gilson C, Jones RJ *et al*: **Abiraterone for prostate cancer not previously treated with hormone therapy**. *New England Journal of Medicine* 2017, **377**(4):338-351.

7. Fizazi K, Tran N, Fein L, Matsubara N, Rodriguez-Antolin A, Alekseev BY, Özgüroğlu M, Ye D, Feyerabend S, Protheroe A *et al*: **Abiraterone plus Prednisone in Metastatic, Castration-Sensitive Prostate Cancer**. *N Engl J Med* 2017, **377**(4):352-360.

8. Chi KN, Agarwal N, Bjartell A, Chung BH, Gomes A, Given R, Soto AJ, Merseburger AS, Ozguroglu M, Uemura H *et al*: **Apalutamide for Metastatic, Castration-Sensitive Prostate Cancer**. *New England Journal of Medicine* 2019, **381**(1):13-24.

9. Fizazi K, Foulon S, Carles J, Roubaud G, McDermott R, Fléchon A, Tombal B, Supiot S, Berthold D, Ronchin P *et al*: **Abiraterone plus prednisone added to androgen deprivation therapy and docetaxel in de novo metastatic castration-sensitive prostate cancer (PEACE-1): a multicentre, open-label, randomised, phase 3 study with a 2 × 2 factorial design**. *The Lancet* 2022, **399**(10336):1695-1707.

10. Smith MR, Hussain M, Saad F, Fizazi K, Sternberg CN, Crawford ED, Kopyltsov E, Park CH, Alekseev B, Montesa-Pino Á *et al*: **Darolutamide and Survival in Metastatic, Hormone-Sensitive Prostate Cancer**. *N Engl J Med* 2022, **386**(12):1132-1142.
