# Supplementary material for: Research Protocol for an Observational Health Data Analysis on the Adverse Events of Systemic Treatment in Patients with Metastatic Hormone-sensitive Prostate Cancer: Big Data Analytics Using the PIONEER Platform
Source: Eur Urol Open Sci. 2024 Mar 25;63:81–8. doi: 10.1016/j.euros.2024.02.019 (PMC10987796; doi:10.1016/j.euros.2024.02.019)
Supplement: Supplementary data 4 [file mmc4.docx]

|  | Indication |  | FDA approval | EMA approval |
| --- | --- | --- | --- | --- |
| Abiraterone^1,2^ | mHSPC |  | 2018 | 2017 |
| Abiraterone^3,4^ | mCRPC |  | 2011 | 2011 |
| Apalutamide^5,6^ | mHSPC |  | 2019 | 2020 |
| Apalutamide^7,8^ | nmCPRC |  | 2018 | 2018 |
| Darolutamide^9,10^ | mHSPC |  | 2022 | 2023 |
| Darolutamide^11, 12^ | nmCPRC |  | 2019 | 2020 |
| Docetaxel^13^ | mHSPC |  | 2015* | |
| Docetaxel^14,15^ | mCRPC |  | 2004 | 2004 |
| Enzalutamide^16,17^ | mHSPC |  | 2019 | 2021 |
| Enzalutamide^18,19^ | mCRPC |  | 2012 | 2013 |
| Enzalutamide^20,21^ | nmCRPC |  | 2018 | 2018 |

**Supplementary Table 4.** Drug of interest approvals

*Level 1 evidence available, but not approved

Abbrviations: EMA; FDA; nmCRPC; mCRPC; mHSPC

**1.** <https://www.fda.gov/drugs/resources-information-approved-drugs/fda-approves-abiraterone-acetate-combination-prednisone-high-risk-metastatic-castration-sensitive> **2.** <https://www.onclive.com/view/eu-approves-abiraterone-for-newly-diagnosed-hormonesensitive-prostate-cancer> **3.** <https://www.fda.gov/drugs/resources-information-approved-drugs/2011-notifications> **4.** <https://www.ema.europa.eu/en/medicines/human/EPAR/zytiga#ema-inpage-item-authorisation-details> **5.** <https://www.fda.gov/drugs/resources-information-approved-drugs/fda-approves-apalutamide-metastatic-castration-sensitive-prostate-cancer> **6.** <https://bwnews.pr/2uFsKH> **7.**<https://www.pharmacytimes.com/view/erleada-for-prostate-cancer> **8.** <https://www.jnj.com/janssen-receives-positive-chmp-opinion-for-erleada-apalutamide-for-patients-with-non-metastatic-castration-resistant-prostate-cancer-who-are-at-high-risk-of-developing-metastatic-disease> **9.** [https://www.fda.gov/drugs/resources-information-approved-drugs/fda-approves-darolutamide-tablets-metastatic-hormone-sensitive-prostate-cancer](https://www.fda.gov/drugs/resources-information-approved-drugs/fda-approves-darolutamide-tablets-metastatic-hormone-sensitive-prostate-cancer%20)  **10.** <https://www.esmo.org/oncology-news/ema-recommends-extension-of-indications-for-darolutamide> **11.** <https://www.fda.gov/drugs/resources-information-approved-drugs/fda-approves-darolutamide-non-metastatic-castration-resistant-prostate-cancer> **12.** <https://www.ema.europa.eu/en/medicines/human/EPAR/nubeqa#ema-inpage-item-authorisation-details> **13.** <https://www.thelancet.com/journals/lanonc/article/PIIS1470-2045(15)00489-1/fulltext> **14.** <https://www.accessdata.fda.gov/drugsatfda_docs/nda/2004/20-449s028_Taxotere.cfm> **15.** <https://link.springer.com/article/10.2165/00128413-200414630-00051> **16.** <https://www.fda.gov/drugs/resources-information-approved-drugs/fda-approves-enzalutamide-metastatic-castration-sensitive-prostate-cancer> **17.** <https://www.esmo.org/oncology-news/ema-recommends-extension-of-indications-for-enzalutamide> 18. [https://www.cancernetwork.com/view/fda-approves-enzalutamide-xtandi-late-stage-prostate-cancer](https://www.cancernetwork.com/view/fda-approves-enzalutamide-xtandi-late-stage-prostate-cancer%2019). **19.** <https://www.europeanpharmaceuticalreview.com/news/28537/xtandi-enzalutamide-now-approved-europe-treatment-men-metastatic-castration-resistant-prostate-cancer-chemotherapy-naive/> 20. <https://www.fda.gov/drugs/resources-information-approved-drugs/fda-approves-enzalutamide-castration-resistant-prostate-cancer> **21.** <https://www.astellas.com/en/news/14311>
